# Epigenetic inheritance of proteostasis and ageing

**DOI:** 10.1042/EBC20160025

**Published:** 2016-10-15

**Authors:** Cheryl Li, Olivia Casanueva

**Affiliations:** ^1^ Epigenetics Programme, The Babraham Institute, Cambridge, CB22 3AT, U.K.

**Keywords:** ageing, epigenetics, proteostasis, transgenerational epigenetic inheritance

## Abstract

Abundant evidence shows that the genome is not as static as once thought and that gene expression can be reversibly modulated by the environment. In some cases, these changes can be transmitted to the next generation even if the environment has reverted. Such transgenerational epigenetic inheritance requires that information be stored in the germline in response to exogenous stressors. One of the most elusive questions in the field of epigenetic inheritance is the identity of such inherited factor(s). Answering this question would allow us to understand how the environment can shape human populations for multiple generations and may help to explain the rapid rise in obesity and neurodegenerative diseases in modern society. It will also provide clues on how we might be able to reprogramme the epigenome to prevent transmission of detrimental phenotypes and identify individuals who might be at increased risk of disease. In this article, we aim to review recent developments in this field, focusing on research conducted mostly in the nematode *Caenorhabditis elegans* and mice, that link environmental modulators with the transgenerational inheritance of phenotypes that affect protein-folding homoeostasis and ageing.

## Introduction

### Proteostasis and ageing

Protein homoeostasis (proteostasis) has been defined as the state in which the proteome is kept in functional balance in an organism [1]. Maintaining proteostasis requires a network of cellular functions including protein synthesis, degradation and folding [1]. An important function of these systems is to prevent the potentially toxic accumulation of misfolded and aggregated proteins [1,2]. Cellular stress responses are conserved transcriptional programmes that induce the expression of genes, including chaperones, that function to rebalance proteostasis in response to harsh environmental conditions [3]. Stress responses become compromised as a function of age in invertebrate model organisms such as the nematode *Caenorhabditis elegans* [2,4] as well as in senescent fibroblasts and tissues from old organisms in different species up to monkeys [5–7]. In old individuals, both protein synthesis and degradation are dysregulated, leading to accumulation of protein aggregates [8–10]. Proteostasis collapse and the accumulation of proteotoxic aggregates is also a key signature of age-related human NDs (neurodegenerative diseases) including HD (Huntington's disease), ALS (amyotropic lateral sclerosis) and MJD (Machado–Joseph disease) [2,11]. Pharmacological and genetic manipulations involving networks of chaperones affect ND models [12]. The importance of molecular chaperones in maintaining neuronal proteostasis in humans is highlighted further by the identification of mutations in molecular chaperones in familial cases of ND [12]. These observations suggest that the loss of proteostasis in neurons is a common feature of the ageing process and may drive the appearance of ageing-related disorders in humans.

Ageing is a complex process defined by progressive functional deterioration and eventual loss of viability [13]. Both genetic and environmental components interact to modulate lifespan [13]. For example, most genetically encoded longevity pathways, such as insulin signalling and target of rapamycin, have in common the capacity to respond to environmental cues by altering both metabolic programmes as well as proteostasis maintenance pathways including stress responses [13]. Dietary restriction is a known environmental intervention that extends lifespan by triggering an adaptive shift of energy resources towards anabolism and somatic maintenance with beneficial effects for proteostasis and health [14]. In addition to the immediate effects on the organism, evidence now shows that some environmental inputs, including stress and nutrition, can cause heritable changes in gene expression that contribute to an offspring's health and lifespan for multiple generations [15,16]. In this article, we summarize recent evidence obtained from model organisms, particularly *C. elegans* and mice, showing that the inheritance of non-genetic components can contribute to proteostasis-related phenotypes across generations. Understanding how the environment can contribute to these phenotypes across generations is especially important for public health with the increasing lifespan of humans in the modern age.

### Chromatin and small RNA modulation and its effect on gene transcription

**Figure 1:**
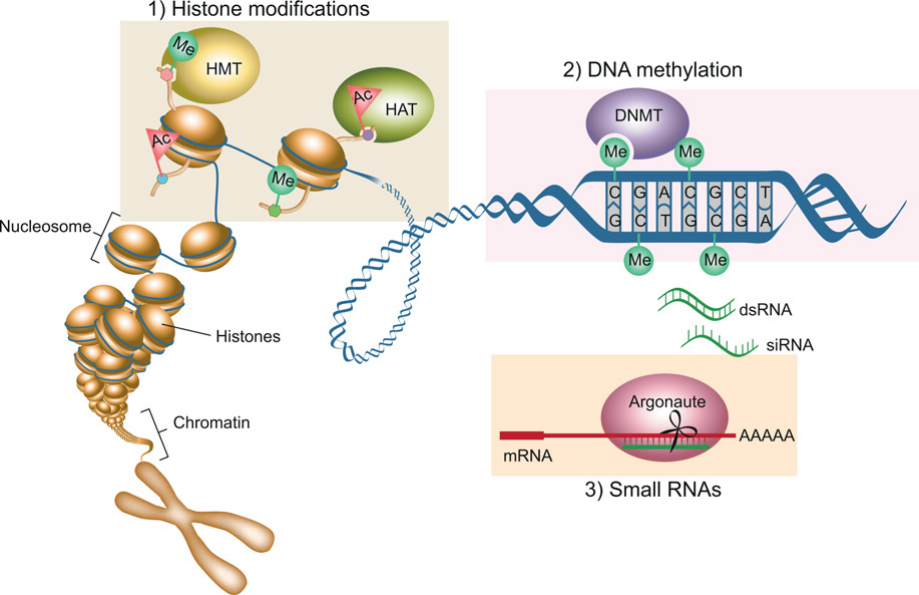
Mechanisms of epigenetic gene regulation In its native state, DNA is wrapped around histone proteins, compacting DNA into chromatin, folded further to form chromosomes. (**1**) Chemical modifications catalysed by enzymes such as histone methyltransferase (HMT) or histone acetyltransferase (HAT) result in modifications on the N-terminal tails of histone proteins can control whether the chromatin is tightly packaged and silent or loosely packaged and actively expressed. (**2**) Another enzymatic epigenetic modification is methylation of DNA on cytosines that packages DNA into silent chromatin. (**3**) Small RNAs (generated by transcription from DNA or exogenously injected dsRNAs) are bound by Argonaute proteins and used as guides to find complementary mRNA sequences. The Argonaute protein can then cause gene silencing by cleaving the mRNA.

Epigenetic mechanisms regulate chromatin structure, allowing the DNA to be organized and compacted into the nucleus in a way that permits appropriate gene transcription and silencing [17]. The three main mechanisms of epigenetic gene regulation include histone modifications, DNA methylation and small RNAs. In the nucleus, DNA is maintained as a highly condensed structure called chromatin. The main subunit of chromatin, the nucleosome, consists of 147 base pairs of DNA wrapped around an octamer of histone proteins (two copies of each of histones H2A, H2B, H3 and H4) [18]. Specialized histones within nucleosomes or chemical modifications of the histones and/or DNA correlate with the ability or inability of chromatin to form higher-order structures, which directly influences transcriptional output (Figure 1).

Actively transcribed genes are characterized by a lack of DNA methylation and activating histone modifications such as methylation of Lys^4^ of histone H3 (H3K4me), which promotes an open chromatin configuration called euchromatin [18]. In contrast, repressed genes are characterized by DNA methylation and silencing histone marks, such as those methylated on Lys^27^ or Lys^9^ (H3K27 and H3K9), which promote a condensed chromatin configuration called heterochromatin [18]. In addition, Polycomb group and TrxG (Trithorax group) proteins play important roles in promoting the stable and heritable repression and activation of gene expression respectively [19]. Finally, epigenetic gene regulation can involve RNAi, which operates through dsRNA and the generation of siRNAs [20]. siRNAs include miRNAs, piRNAs (PIWI-interacting RNAs) and endo-siRNAs (endogenous siRNAs) which can silence gene expression through a variety of mechanisms [20]. In *Schizosaccharomyces pombe*, *Arabidopsis thaliana* and *C. elegans*, siRNAs can act as sequence-specific guides to direct enzymes that establish silencing histone marks on to chromatin and aid in heterochromatin formation [20,21] (Figure 1).

### Heritable epigenetic modifications

**Figure 2:**
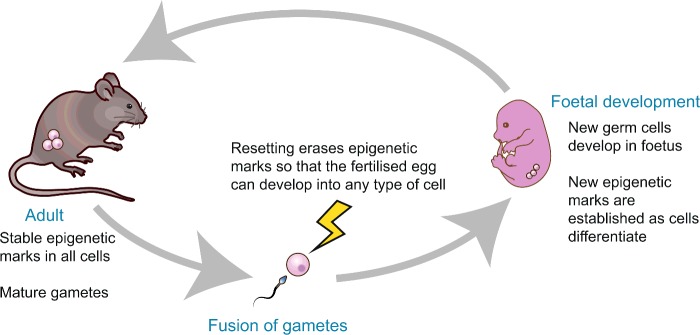
Reprogramming erases epigenetic marks During gametogenesis and early development, a process called germline resetting takes place, where most epigenetic marks in chromatin are removed. This allows the reacquisition of pluripotency in the early embryo and proper differentiation of tissues during development.

Epigenetics is thought to be an important integrator between the environment and gene expression. For example, early life nutrition can directly programme epigenetic marks throughout the genome (the ‘epigenome’), resulting in long-term effects on gene expression and physiology throughout life [17]. August Weismann formulated the germ-plasm theory of heredity, stipulating that only germ cells can pass on genetic information to the next generation, whereas somatic cells have no inheritance function [22]. The idea that genetic information cannot pass from the soma to the germline and on to the next generation is known as the Weismann barrier [22]. Two key questions in the field are whether environmentally induced epigenetic changes in the germline can be inherited in the offspring and whether the Weisman barrier can be crossed. Epigenetic changes that occur during the lifetime of an individual's germline are not generally thought to be inherited into the next generation, as both histone and DNA modifications are erased and re-established in each generation through a developmental reprogramming process that resets the epigenome of the zygote at fertilization to renew totipotency [23] (Figure 2). However, there is evidence that at least some epigenetic modifications can escape this reprogramming, a notable example being the incomplete erasure of DNA methylation at the *A^vy^* locus in mice [24]. Likewise, new evidence suggests that there are some exceptions to the Weisman theory of heredity [15,16].

**Figure 3:**
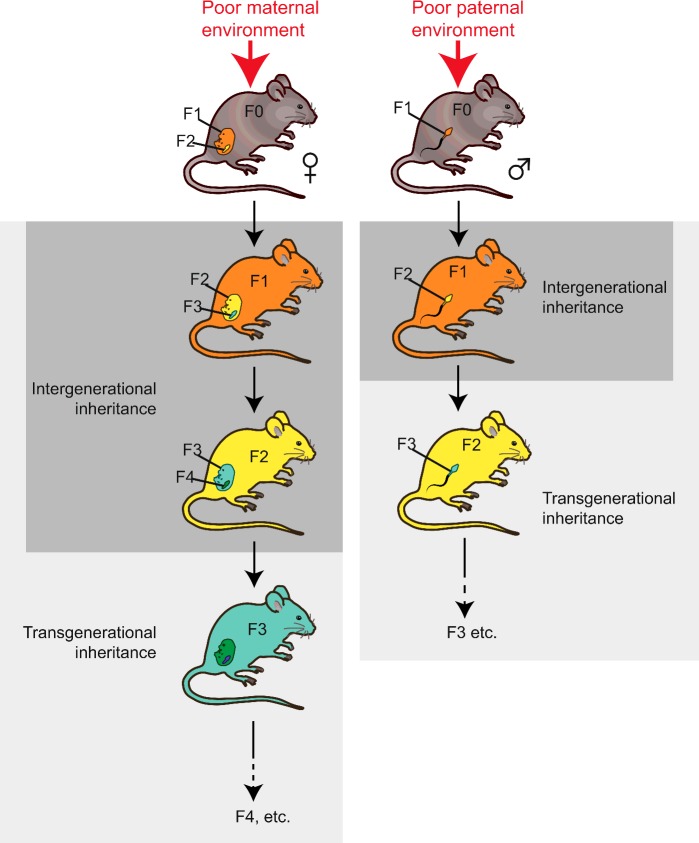
Intergenerational and transgenerational inheritance Maternal exposure to a poor environment exposes both her developing fetus *in utero* (F1, orange) as well as the developing germ cells in the fetus which later give rise to F2 (yellow), both of which are present at the time of environmental exposure (referred to as inter-generational inheritance). However, the cells which form the F3 generation (green) are not present at the time of environmental exposure; if they carry phenotypes, they do so by transgenerational inheritance. For paternal exposure to poor environment, only the sperm cells which form the F1 generation are present and directly exposed to the poor environment (intergenerational inheritance), whereas phenotypes which persist into F2 and beyond are referred to as transgenerational inheritance.

Exposure of an organism to environmental stressors can affect offspring health for one generation (immediate children only), two generations or three or more generations (Figure 3). When the exposure is maternal, the first generation offspring (F1) are directly exposed to the environment as fetuses *in utero*. In addition, the fetus's germ cells (which will give rise to the F2) are also subjected to the stressor, because they are already present and developing while *in utero*. In contrast, paternal exposure only results in direct exposure of the male's sperm. In this situation, only the cells which give rise to the F1 are directly exposed, but not the F2. The mode of inheritance that only affects generations that were directly exposed to stressors is referred to as inter-generational inheritance. This includes parental-effect phenotypes, such as passage of RNA molecules and maternal proteins from oocytes to embryos, or it can be mediated by chromatin changes in the exposed fetuses or germ cells that are not inherited further. When altered offspring phenotypes persist into generations which were never exposed to parental stress, however, it can be inferred that there must have been induction of a stable epigenetic change which persists through epigenetic resetting and is transmitted to the newly formed germ cells; this is referred to as transgenerational epigenetic inheritance. For chromatin marks involving histones or DNA methylation, the transgenerational effects are generally short-lived, lasting three or four generations (depending on the parent of origin) [26,27]. Small RNA-mediated inheritance is also short-lived, but in some rare cases it has been shown to be heritable for up to 80 generations [20].

## Inter- and trans-generational inheritance of lifespan and proteostasis-related phenotypes

### Evidence from invertebrate model organisms

Invertebrate model organisms such as the nematode *C. elegans* and the fruitfly *Drosophila melanogaster* have been quite useful in the study of transgenerational inheritance owing to their short generation times and the many genetic tools available. Epigenetic regulation in invertebrates relies mostly on histones and small RNA-mediated pathways because DNA methylation is either absent or not highly represented [28]. In *D. melanogaster*, it has been demonstrated that paternal exposure to high dietary sugar evokes an increase in triacylglycerol storage in the next generation, which results from heritable H3K9/K27 histone modifications in the sperm [25]. Although transgenerational proteostasis-related phenotypes were not reported, maternal exposure to high dietary glucose in *C. elegans* is known to be protective against cellular stress and also reduces age-dependent proteotoxicity in the next generation, as is evident by the delayed onset of neurodegeneration in an ALS model [29]. This intergenerational inheritance was dependent on H3K4me3 marks, but it is not clear whether the marks themselves are inherited or whether alternative mechanisms are in place.

Nutrition is not the only stressor with heritable consequences for proteostasis. In *C. elegans*, heat shock activates the transcriptional up-regulation of chaperones that function to refold the unbalanced proteome [3]. *In vivo* transcriptional reporters for chaperones have revealed widespread inter-individual variability in expression among nematode worms which are genetically identical [30,31], and this variability has been shown to have important fitness consequences for lifespan, stress resistance and mutation penetrance [30,31]. Importantly, the overall level of expression in individual worms is inter-generationally inherited, such that worms expressing more *hsp16.2* transcript give rise to progeny with more transcript and so forth [32]. The mechanism underlying this inter-generational memory of heat stress and its potential consequences for the next generation remains unexplored, but might involve epigenetic mechanisms. Exposure to heat stress is known to alter the epigenome; for example, heat shock or osmotic stress during early *Drosophila* development can disrupt heterochromatin for several generations, even after the stressors have been removed, albeit without any noticeable additional phenotypic consequences [33]. These examples point towards widespread contributions of environmental stress and nutrition that can be inherited across generations in invertebrates.

**Figure 4:**
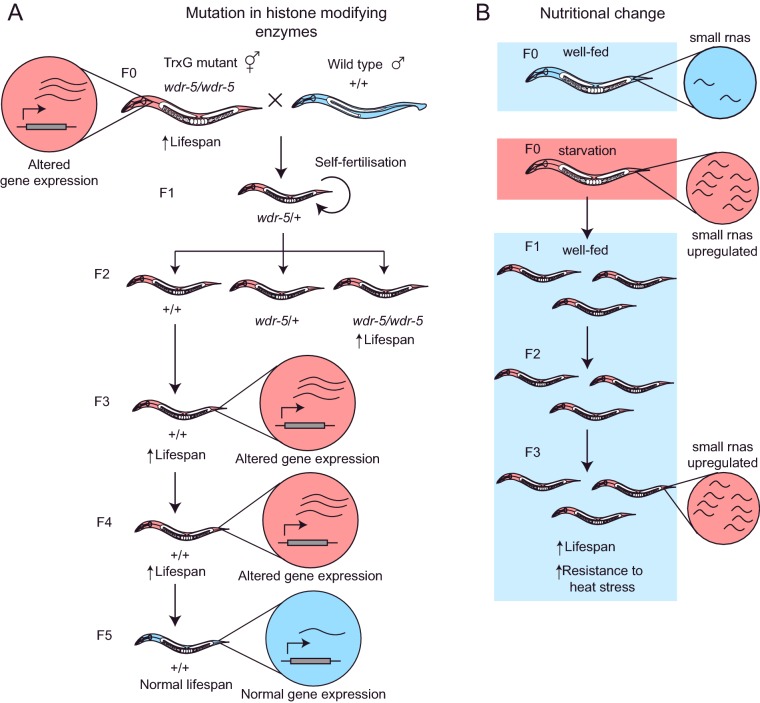
Examples of transgenerational epigenetic inheritance in invertebrate model systems (**A**) Mutations in the histone-modifying enzyme WDR-5 (which forms part of the TrxG complex) causes increased longevity in *C. elegans*. When *wdr-5* mutants are crossed with wild-type worms and their heterozygous F1 progeny selfed to produce wild-type (+/+) offspring, the F3 and F4 progeny are also long-lived and exhibit gene expression changes similar to the *wdr-5* mutant, despite lacking the mutation themselves. This inherited change is not perpetuated in the F5 offspring, however, suggesting that the epigenetic changes are removed after three generations. (**B**) Starvation also induces upregulation of small RNAs in *C. elegans*, which is heritably maintained in F3 offspring despite being returned to normal nutrition for three generations. F3 offspring of starved animals have also been reported to exhibit an increase in lifespan and resistance to heat shock, which may be mediated by these changes in small RNA expression. Red circles represent altered gene expression, and blue circles represent wild-type levels of gene expression. See 15,16,26 for details.

A great deal of evidence for transgenerational epigenetic inheritance has also been found using genetic mutations that deplete chromatin marks [34], and in some cases these marks appear to carry information that influences ageing. For example, in *C. elegans*, mutations in TrxG proteins cause a reduction in H3K4me3 mark deposition and extended lifespan [35]. Moreover, reducing TrxG function in one generation causes heritable lifespan extension for three generations even if normal gene function is restored in the F1 progeny [26] (Figure 4A). These results suggest that there is an epigenetic memory left by the lack of TrxG function that continues to have phenotypic consequences over several generations. Global levels of H3K4me3 are unchanged in wild-type descendants of TrxG mutants, however, indicating that, at least on a gross level, the marks themselves are not heritable. However, a small number of genes with metabolic functions are aberrantly expressed in genetically wild-type descendants in the fourth (but not fifth) generation [26]. Overall, these studies indicate that TrxG activity can mediate a transgenerational epigenetic memory of gene expression, although the inheritance mechanisms responsible for the transgenerational phenotype remain unknown. An important question is whether the environment can trigger transgenerational inheritance of phenotypes in wild-type animals. As mentioned above, in *C. elegans*, glucose can trigger memory through the H3K4 trimethylation complex only on a short inter-generational timescale, therefore it is unknown whether other environmental triggers can cause histone-mediated long-term transgenerational memory.

Finally, invertebrate model organisms have been used to discover that siRNA inheritance can persist for generations after its initial induction from both paternal and maternal sources [36,37]. Currently, the strongest evidence for small RNA inheritance involves the response either to artificially introduced RNA or to mobile genetic elements. So the question remains whether this form of inheritance is only limited to defence against exogenous nucleic acids or whether it also represents a general adaptive phenomenon [20]. Although it is well established that there is transgenerational inheritance mediated by siRNAs [38–40], new studies suggest that siRNAs that are produced in the soma in response to the environmental stimuli can be inherited through the germline across generations, violating the Weisman's germline/soma barrier. For example, in *C. elegans*, early life starvation relays heritable information that extends lifespan for up to four generations, even if the progeny are exposed to a normal food supply [15] (Figure 4B). This inheritance depends on endo-siRNA pathways [15]. However, the extent of lifespan inheritance in this study was highly variable among biological replicates, and in a later study only the animals that were most severely affected by early larval starvation showed reliable transgenerational inheritance of stress resistance [16]. Carefully controlled mechanistic analysis will further clarify the role of siRNAs on transgenerational inheritance of stress resistance and ageing.

### Evidence of inter- and trans-generational inheritance of proteostasis-related phenotypes in mammals

**Figure 5:**
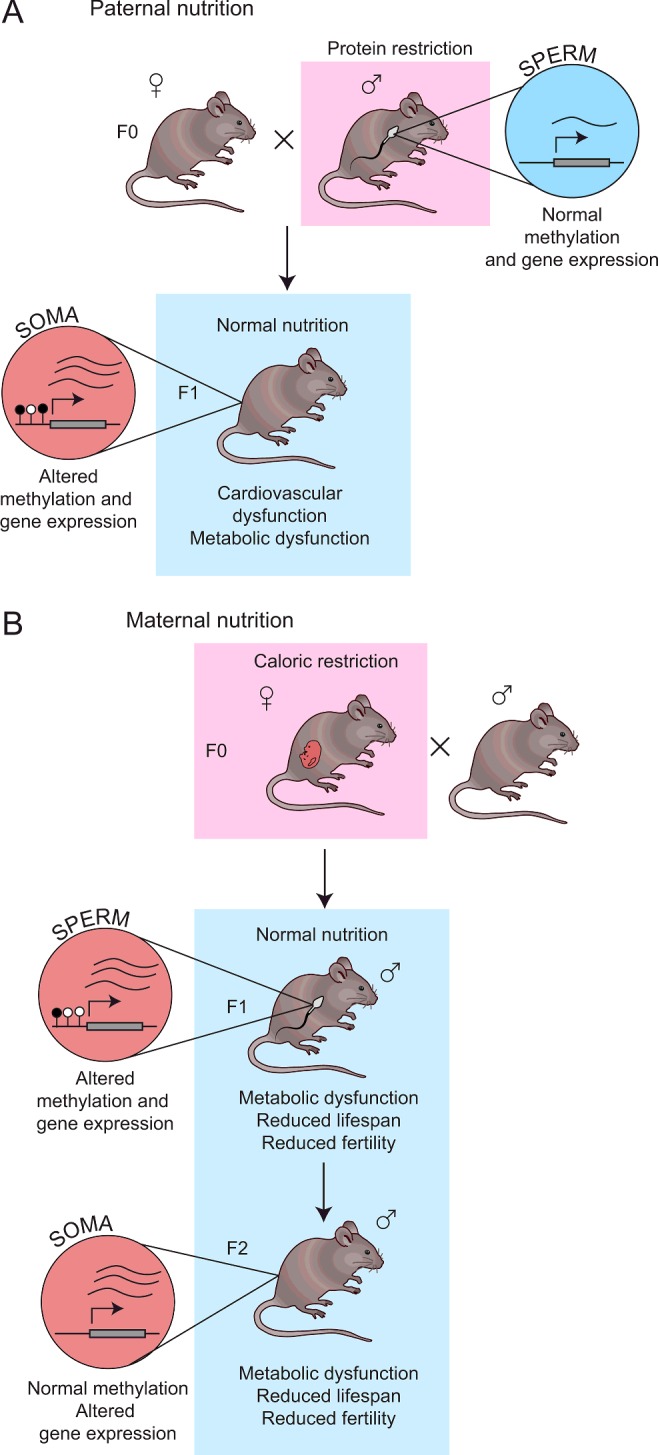
Examples of intergenerational epigenetic inheritance in vertebrate model systems (**A**) Paternal protein restriction has been reported to cause intergenerational inheritance of metabolic symptoms in F1 offspring, despite being restored to normal nutrition postnatally. Analysis of liver tissue in F1 offspring of protein-restricted fathers showed altered DNA methylation and gene expression changes, which may contribute to the phenotype. No altered methylation was detected in the sperm of protein-restricted fathers, however. (**B**) Maternal caloric restriction causes metabolic dysfunction, reduced lifespan and fertility in offspring up to the F2 generation. Analysis of sperm in F1 offspring revealed methylation and gene expression changes which may be linked to the phenotype. Gene expression changes were also observed in the brains and livers of F2 offspring, but no methylation changes were observed. Red circles represent altered gene expression, and blue circles represent wild-type levels of gene expression. Figure references: [42,45,62,64].

Is transgenerational inheritance of ageing and proteostasis-related phenotypes conserved in more complex organisms? Most studies have focused on metabolism and other health-related phenotypes with only a few studies focused on late-onset phenotypes such as ageing and proteostasis, probably due to the long generational time of mammalian models. In humans, the most important epidemiological studies have been carried out in populations exposed to famine and strict food rationing during wartime situations [41]. These studies show that poor maternal nutrition during gestation is linked to numerous effects on offspring health, ranging from glucose tolerance, insulin resistance and diabetes to increased adiposity and body size, risk of cardiovascular disease, obesity and schizophrenia [41]. In both humans and rodents, these effects have been shown to be intergenerationally inherited in grandchildren (F2), even if the children (F1) themselves are not malnourished [42–44] (Figure 5). Paternal models of nutrient restriction in mice can also produce similar intergenerational effects in offspring [45]. It is thought that poor parental nutrition can ‘programme’ the offspring's metabolism in preparation for poor postnatal nutrition, but that these metabolic changes may also inadvertently predispose offspring to disease when they are born in conditions of normal abundant nutrition [46].

It is interesting that the intergenerational changes seen in offspring typically include an increased predisposition to late-onset diseases that are associated with ageing and loss of proteostasis. In fact, several studies in rats show that offspring born to mothers fed on a low-protein diet during pregnancy have shorter lifespans [47–49] and become less fertile at a younger age [49–51] than those fed on a normal diet. Moreover, recent studies suggest that maternal nutrient restriction may also result in reduced F2 offspring lifespan [52] and accelerated reproductive ageing [53]. This accelerated ageing and loss of fertility may be linked to increased oxidative stress [51,54], which is known to contribute to ageing. New studies are planned to address the possibility of an accelerated ageing phenotype in humans in response to maternal nutrition [55]. Most of the aforementioned studies have addressed inter-generational memory of phenotypes and very few studies have assessed whether environmentally induced transgenerational inheritance of phenotypes persists beyond F2. The evidence is still in some cases contradictory, with some studies reporting only intergenerational inheritance [56,57], whereas others report transgenerational inheritance [27].

Inter- and trans-generational inheritance of increased propensity to NDs has not yet been reported, although epigenetic changes are known to be associated with neurodegeneration [58]. Furthermore, NDs have many known environmental risk factors and there is evidence that early life exposure can increase the risk of neurodegeneration later on in life, similar to the effects of maternal nutrition [59]. Tentative evidence that ND might affect more than one generation comes from the observation that inhibitors of histone-modifying enzymes in an HD mouse model can cause DNA methylation changes that are inherited and associated with improved disease phenotype [60]. This is an area of research that is likely to grow and yield interesting insights in the future.

### Mechanisms of epigenetic inheritance via DNA methylation in mammals

In contrast with invertebrate studies, most mammalian work has focused on changes in DNA methylation, which have historically been easier to assay and interpret than changes in histone modifications. New technologies have now enabled a genome-wide view of DNA methylation in mammalian models of intergenerational inheritance, with several studies revealing many changes in both F1 and F2 offspring [61–64]. A very important consideration is whether these changes are functional and have an effect on gene expression. In some cases, the methylation changes do appear to be functional: Carone et al. [62] reported that in offspring of protein-restricted fathers the DNA hypermethylation at *Ppara*, a gene encoding peroxisome-proliferator-activated receptor α, a transcription factor with key roles in lipid metabolism, accurately reflected a corresponding decrease in gene expression [62]. Similarly, methylation changes in insulin signalling pathway genes were found to correspond with expression changes in offspring of diabetic high-fat-diet-fed fathers [63]. However, overall DNA methylation changes detected in these studies did not globally correlate with changes in gene expression of nearby genes. This suggests that, even if methylation changes occur, they may not always consistently affect expression of their presumed downstream targets. Alternatively, depending on the spatial organization of the genome, they may be affecting expression of distant genes.

In order to determine whether epigenetic changes in the offspring are indeed the consequence of inter- or trans-generational epigenetic inheritance mechanisms, the same altered methylation pattern should be detected in germ cells from exposed parents and in their offspring. In the aforementioned study, methylation of the *Ppara* gene was not altered in the sperm of fathers exposed to protein restriction, despite *Ppara* being aberrantly hypermethylated in their offspring [62]. Similarly, Radford et al. [64] reported that altered methylation patterns in the sperm of fathers exposed to maternal caloric restriction did not persist in F2 offspring, although altered expression of these genes still persisted in F2 despite the absence of any methylation change. This discontinuity in DNA methylation suggests that inter- and trans-generational epigenetic inheritance does not occur through inheritance of altered DNA methylation marks through the germline, but by some other epigenetic mechanism (such as small RNAs or histone modifications). Other studies, however, make a strong case for the hypothesis that transgenerational epigenetic inheritance may occur through persistence of DNA methylation marks in the paternal germline: Wei et al. [63] reported that over one-third of differentially methylated loci in the sperm of pre-diabetic male mice fed on a high-fat diet were also differentially methylated in their F1 and F2 offspring [63]. The reasons for conflicting results from the previous two studies are not yet clear. Perhaps the mechanism of transgenerational inheritance for undernutrition is different from that of overnutrition, or discrepancies may simply be due to differences in the techniques and models used. It is likely that additional epigenetic mechanisms are also involved; in particular, Carone et al. [62] reported some H3K27me3 changes in sperm from protein-restricted fathers. Further studies should help to resolve discrepancies in the field.

## Conclusions and discussion

Overall, there is evidence to support the view that epigenetic activation/inactivation of genes does occur in individuals whose ancestors were exposed to challenging environmental conditions, compromising health and lifespan in descendants. The underlying mechanisms are not clear, but when parents are exposed to nutritional stress, the descendants usually show changes in the expression of genes associated with metabolic functions. A known fundamental function of all longevity pathways is to promote lifespan-extending metabolic shifts. Therefore a likely possibility is that the epigenetic memory activated in the ancestors targets at least part of these pro-longevity metabolic pathways. Only a few studies have directly investigated transgenerational inheritance in the context of proteostasis. Do all interventions that prolong lifespan also reduce proteotoxicity? Most available evidence is consistent with the view that the ageing process actively reduces the cell's ability to detoxify small toxic aggregates, and therefore, in principle, all interventions that extend lifespan should also reduce proteotoxicity. In fact, any intervention that alters basic cellular functions related to proteostasis, including protein synthesis, degradation or folding, will have an impact, directly or indirectly, on proteotoxicity. Given the paucity of data, future studies should be specifically designed to study the effect of epigenetic inheritance on proteotoxicity and NDs.

For invertebrate models, only transcriptional changes in subsequent generations have been reported, but the epigenetic modifications that lead to these gene expression changes have not been measured directly. Likewise, in vertebrate models, phenotypic, transcriptional and DNA methylation evidence has been found in the offspring, but the precise mechanism remains elusive, and very little research has been conducted on other epigenetic mechanisms such as histone modifications. The use of new and improved technologies for single cell epigenomics should facilitate the identification of the heritable DNA modifications that lead to gene regulatory differences in both invertebrate model organisms and mammals. New studies should also focus on further dissecting the potential role of small RNAs in epigenetic inheritance across species.

The strongest evidence that supports the view that there is heritable non-genetic modulation of gene expression that contributes to proteostasis comes from invertebrate model organisms that lack DNA methylation. In mammals, evidence for transgenerational epigenetic inheritance is less common. It remains possible that the phylogenetic acquisition of DNA methylation co-evolved with more stringent reprogramming events in the germline. Perhaps the passage of information about stress and nutritional conditions is more important in species with short generation times, where environmental changes that affect one generation are also relevant to the next generation. A more mechanistic understanding of reprogramming, and more carefully conducted studies will help to clarify how pervasive these mechanisms are in mammals including humans.

Understanding the mechanisms of transgenerational epigenetic inheritance has many potential beneficial applications: for example, identifying the precise epigenetic marks that are transmitted could allow us to better predict and determine patients at risk of metabolic or proteostasis-related disorders caused by a clinical history of disease in past generations, as well as their risk of disease transmission to future offspring. Furthermore, understanding the fundamental mechanisms underlying transgenerational epigenetic memory and germline reprogramming may eventually make it possible to develop technologies to prevent transmission of programmed disease risk to subsequent generations (for instance by targeting the relevant chromatin-modifying enzymes/small RNAs). Lastly, understanding the mechanisms of germline epigenetic reprogramming may also help us to better understand and improve the reprogramming of somatic cells for cloning and other translational research. The prospect of regenerative and preventive medicine calls for a more thorough understanding of the epigenome as cells age as well as the potential contribution of past generations.

## Summary

- Dysregulation of proteostasis an important factor in ageing and disease.
- Environmental stressors may impact gene expression and health for multiple generations through changes in epigenetic gene regulation.
- Both proteostasis-associated phenotypes and epigenetic changes have been observed to persist for more than one generation in both invertebrate and vertebrate model organisms in response to environmental stressors; a summary of current research in the field is presented.
- We speculate on the possibility of transgenerational epigenetic inheritance of proteostasis and key prospective research directions.

## Abbreviations:

ALS: amyotropic lateral sclerosis
endo-siRNA: endogenous siRNA
HD: Huntington's disease
ND: neurodegenerative disease
TrxG: Trithorax group
